# Comparison of outcomes between flexible ureteroscopy and mini-percutaneous nephrolithotomy in the management of upper calyceal calculi larger than 2 cm

**DOI:** 10.1186/s12894-022-01142-0

**Published:** 2022-11-15

**Authors:** Hanqing Xuan, Zhebin Du, Lei Xia, Yang Cao, Qi Chen, Wei Xue

**Affiliations:** Department of Urology, Renji Hospital, School of Medicine, Shanghai Jiao Tong University, 1630 Dongfang Road, Pudong District, Shanghai, 200127 People’s Republic of China

**Keywords:** Upper calyceal calculi, Calculi > 2 cm, F-URSL, m-PCNL

## Abstract

**Objective:**

To compare the outcomes of FURSL and m-PNL in the management of upper calyceal calculi larger than 2 cm.

**Methods:**

A total of 75 patients with upper calyceal calculi larger than 2 cm that were treated by FURSL (n = 37) or mini-PNL (n = 38) were retrospectively analysed. The mean age, sex, stone burden, operative time, complications, length of hospitalization, and stone-free rate (SFR) were compared between the groups. The success of the procedure was defined by the absence of residual stones or residual fragments smaller than 4 mm on computed tomography at 4 weeks postoperatively.

**Results:**

The two groups had comparable preoperative parameters. The mean operative time was significantly longer in the mini-PNL group than in the FURSL group (87.8 vs. 69.8 min, *p* < 0.001). The length of hospitalization was greater in the mini-PNL group than in the FURSL group (2.5 vs. 1.3 days, *p* < 0.001). Although the perioperative complication rate was higher in the mini-PNL group (23.7%) than in the FURSL group (13.5%), this difference was not statistically significant (*p* = 0.258). The SFR for the mini-PNL group was 89.5%, and that of the FURSL group was 81.1%; the difference was not significantly different (*p* = 0.304).

**Conclusions:**

Both FURSL and mini-PNL are effective and safe for the management of upper calyceal calculi larger than 2 cm. Of these two procedures, mini-PNL is less time consuming, FURSL is associated with faster recovery. FURSL can be considered a good alternative treatment in selected patients.

## Introduction

The management options for renal calculi include surveillance, medical treatment, extracorporeal shock wave lithotripsy (ESWL), percutaneous nephrolithotomy (PNL), flexible ureteroscopy with holmium laser lithotripsy (flexible ureteroscopy and lasertripsy, FURSL), laparoscopy and open surgery, but treatment practices have changed dramatically over the last few decades, with a shift from open surgery to minimally invasive interventions [1]. ESWL has been recommended as the first-line therapy for renal calculi < 2 cm, while it is conventionally believed that complex stones larger than 2 cm, multiple renal stones, staghorn calculi and stones in unfavourable areas of the kidney (such as the lower pole) are more suitable for PCNL because of its high stone-free rates [2, 3]. However, PNL is more invasive and has higher complication rates than ESWL or ureteroscopy [4].

FURSL has been widely used in the management of upper ureteral and renal stones due to its safety and minimal invasiveness. This procedure is especially suitable for patients with renal stones less than 2 cm and patients with obesity or severe spinal deformity [2, 3]. With the development of flexible ureteroscopic instrumentation and holmium laser lithotripsy as well as the accumulation of surgical experience, FURSL has become an increasingly considered option for complex renal stones larger than 2 cm as an alternative to PNL. However, it remains uncertain which technique is superior. In the present study, we attempted to explore the relative benefits of FURSL and mini-PNL in the management of upper calyceal stones larger than 2 cm.

## Methods

### Patients

A retrospective review was conducted for 75 patients diagnosed with upper calyceal calculi who underwent FURSL and mini-PNL by a single surgeon (Qi C) at Ren Ji Hospital from January 2015 to January 2017. Chart review was used to obtain patient, stone, and treatment parameters. The inclusion criteria were single or multiple renal stones confined to the upper calyces and ranging from 2 to 4 cm in diameter. The exclusion criteria were as follows: patients with concomitant ureteral stones, bladder stones, renal tuberculosis, renal tumour, renal dysfunction, acute or chronic nephritis, nephrotic syndrome, obesity, or pregnancy.

**Fig. 1:**
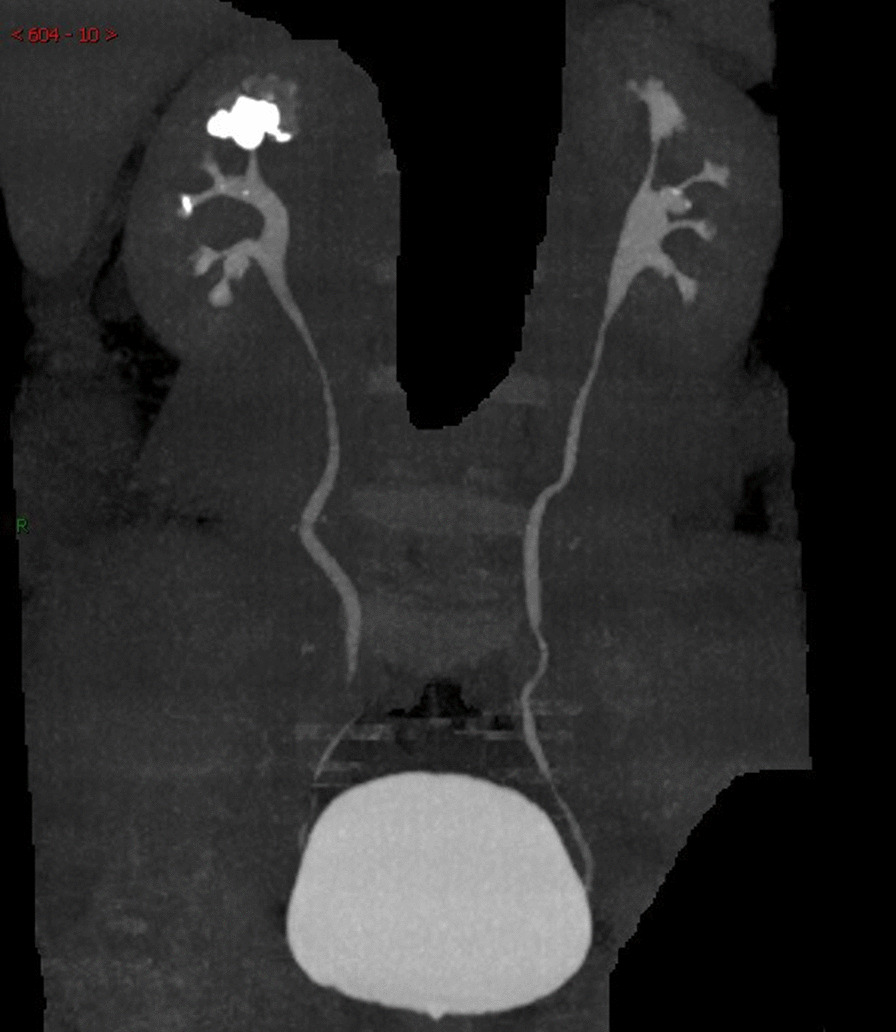
Preoperative CTU showed a 2.5 cm stone located in the upper pole calyceal

Surgical informed consent was obtained and included specific mention of the possible need for multiple ureteroscopic procedures to treat the multiple renal stones, the need for a second-look diagnostic procedure, and stent placement. Each patient underwent a preoperative computed tomography (CT) urogram to determine the anatomy collecting system and the total burden of calculi (measured as the cumulative diameter of the renal stones) (Fig. 1) and to ensure that the stones were not harboured within a diverticulum or the parenchyma of the kidney. Urinary infection was treated preoperatively with antibiotics. A sterile urine culture was obtained from each patient before surgery.

### Surgical intervention

#### Surgical procedures for the FURSL group

Patients in the FURSL group were placed in the dorsal lithotomy position and Trendelenburg position under general anaesthesia, and intravenous antibiotics were given. After rigid ureteroscopy, the double-J stent was pulled out, and a zebra guide wire (0.038 mm) was then directly placed into the renal pelvis. The ureteroscope was then pulled out, and a ureteral access sheath (UAS) (Cook Medical, Bloomington, IN, USA, 12/14For14/16F) was placed. After urine flowed out of the ureteral sheath, the guide wire was pulled out. An F8.5 flexible ureteroscope (Storz FLEX-X^c^, Tuttlingen, Germany; we used the same flexible ureteroscope for every intervention) and a 365 μm or 200 μm holmium laser fibre were subsequently used for treatment. The holmium laser was set at an energy level of 1.5–2.0 J and at a rate of 10–20 Hz. Sustained low-pressure flushing allowed us to optimally visualize and maintain low intra-pelvic pressure. If the lithotripsy process was influenced by breathing, intermittent apnoea was adopted. Basketing of the fragments was deemed necessary only in cases with residual fragments > 2 mm after multiple procedures. When extraction was deemed necessary, a 2.2F zero-tipped nitinol stone basket (Cook Medical, Bloomington, IN, USA) was used. A 6F double-J stent was placed at the end and removed 2 weeks later. The operative time was defined as the time elapsed from insertion of the rigid ureteroscope to the completion of stent placement.

#### Surgical procedures for the mini-PNL group

After general anaesthesia, an open-end 6F ureteric catheter was placed at the beginning of the procedure. Then, the patients were kept in the prone position. The puncture of the target calyx was performed using an 18-G needle under the guidance of ultrasound. The time needed for the insertion of the ureteric catheter, as well as the time needed for patient positioning, was included in the overall operative time. With the outflow of the fluid or urine, a J-tip guide wire was inserted into the renal pelvis. Dilatation of the tract was performed using 10F, 14F, and 18F facial dilators successively, and an 18F peel-away sheath was inserted. A rigid 13F renoscope was introduced, and stone fragmentation was carried out using a holmium-doped yttrium–aluminium–garnet (Ho:YAG) laser (550 µm fibre; energy 1.5–2.0 J; frequency 15–20 Hz). At the end of the procedure, a 16F nephrostomy tube was left in situ for 1 week. A 6F double-J stent was routinely placed in each patient and removed 2 weeks after the operation. The operative time was defined as the time elapsed from the insertion of ureter catheter via renoscope to fixation of the nephrostomy tube.

The non-contrast CT scan was performed at the fourth week postoperatively, and SFR was evaluated. Stone-free status was defined as the absence of residual stones and residual fragments less than 4 mm. FURSL was repeated if there was any residual fragment larger than 4 mm. Postoperative fever was defined as body temperature > 38 °C.

### Statistical analysis

The SPSS statistical software package was used to analyze the data (v25.0; IBM, US). Continuous variables were expressed as mean and SD, whereas categorical variables were expressed as percentages. A Student's t test or a Mann–Whitney U test were used to examine continuous variables. The categorical variables were compared using the Chi-square test and Fisher's exact test. The statistical significance of a two-tailed P value of 0.05 was determined.

## Results

A total of 75 patients with upper calyceal stones larger than 2 cm were included in the study, with 37 in the FURSL group and 38 in the mini-PNL group. Both groups had comparable preoperative parameters (Table 1). The mean (SD) operative time of the FURSL group was 69.8 ± 13.9 min which was significantly shorter than that of the mini-PNL group (87.8 ± 14.3 min, *p* < 0.001). The mean haemoglobin decrease was 1.2 g/L in the FURSL group and 3.0 g/L in the mini-PNL group (*p* < 0.001); however, no one needed blood transfusion. The hospital stay was longer in the mini-PNL group than in the FURSL group (2.5 vs. 1.3 days, *p* < 0.001). The mean cost of the FURSL group was $2603.80, which was slightly higher than that in the mini-PNL group but without statistical significance (*p* = 0.515). The stone analysis was also conducted in our study, there was no significant difference in the composition of stone between the two patient groups. Both calculus in two procedures was mainly composed of calcium oxalate. (Table 2).

**Table 1:** Demographics data and stone characteristics

Variables	FURSL group	mini-PNL group	*P* value
*Gender*			0.743
Male (n, %)	25 (67.6)	27 (71.1)	
Female (n, %)	12 (32.4)	11 (28.9)	
Age (mean ± SD) (years)	50.6 ± 12.2	51.7 ± 11.2	0.696
No. Left/right side	16/21	23/15	0.134
No. Hypertension (%)	10 (27)	15 (39.5)	0.253
No. Diabetes mellitus (%)	3 (8.1)	5 (13.2)	0.479
*History of surgery*			0.562
Yes	32 (84.2)	31 (81.6)	
NO	5 (15.8)	7 (18.4)	
Mean CT value (HU) (range)	1063 ± 137.5	1140 ± 270.6	0.133
Stone size (mean ± SD) (mm), (range)	28.5 ± 4.5	27.1 ± 4.1	0.69
*Stone number*			0.48
Singe	34 (91.9)	33 (86.8)	
Multiple	3 (8.1)	5 (13.2)

**Table 2:** Perioperative and postoperative data

Variables	FURSL group	mini-PNL group	*P* value
Operation time (mean ± SD) (min)	69.8 ± 13.9	87.8 ± 14.3	0.000
Hospitalization time (mean ± SD) (days)	1.3 ± 0.6	2.5 ± 1.2	0.000
Cost (mean ± SD) ($)	2603.8 ± 516.1	2509.6 ± 711.2	0.515
*Complications, (modified Clavien classification), n (%)*			
*Overall intraoperative*	3 (8.1)	4 (10.5)	0.719
Bleeding (Grade I)	0	3 (7.9)	
Minor pelvic-ureteric injury (Grade I)	3 (8.1)	1 (2.6)	
*Overall postoperative*	2 (5.4)	5 (13.2)	0.249
Fever (Grade I)	2 (5.4)	2 (5.3)	
Gross hematuria (Grade I)	0	3 (7.9)	
Total complications	5 (13.5)	9 (23.7)	0.258
Hemoglobin drop (mean ± SD) (g/L)	1.2 ± 0.7	3.0 ± 1.2	0.000
Stone-free rates (1 month postoperative, %)	81.1	89.5	0.304
*Stone composition*			0.899
Calcium oxalate monohydrate	17	16	
Calcium oxalate dihysdrate	13	12	
Carbonate apatite	5	7	
Uric acid	2	3	

In the FURSL group, there were 3 cases of ureteral mucosa injury and 2 cases of fever, and the incidence of complications was 13.5%. In the mini-PNL group, the major complication was bleeding (6 cases), and there were 2 cases of fever and one case of pelvic mucosa injury. After conservative treatment, all patients' hemoglobin levels were stable at > 8 g/dL, hence no blood transfusions were administered. Although the complication rate was higher in the mini-PNL group, there was no statistically significant difference, as shown in Table 2.

Although the SFR in the FURSL group was 81.1% vs. 89.5% in the PNL group according to the definition of success that was set at the beginning of the study, this difference was not found to be statistically significant. The residual stone’s burden was generally 3-4 mm in our 1-month post-surgery evaluation and there is no need for auxiliary procedures nor readmission. The stone-free status would be re-evaluated 3 months postoperatively in these patients by ultrasound.

## Discussion

The European Association of Urology (EAU) and Chinese Association of Urology (CAU) guidelines on the management of staghorn calculi recommend PNL as a first-line treatment for intrarenal calculi larger than 2 cm and ESWL for renal calculi smaller than 2 cm [2, 3]. Choosing a proper puncture position and a right puncture direction is the key point for PCNL. For most renal stones, the puncture position is usually beneath the twelfth rib or in the eleventh intercostal space from the axillary line to the scapular line, but for upper calyceal stones, it is appropriate to choose the tenth or eleventh intercostal space as the puncture position to shorten the distance between the puncture point and the target renal calyx. It may reduce the possibility of renal parenchyma avulsion due to the movement of the endoscope during surgery. However, it is time consuming to build an operation channel with a higher incidence of intraoperative complications, such as haemorrhage, perforation and urinary sepsis, which are major concerns in clinical practice [5, 6].

With the advent of new generation flexible ureteroscopes with greater deflection and control, FURSL is increasingly used as a primary modality in the management of renal calculi and even for kidney stones > 2 cm with minimal complications and a higher success rate [7]. Pieras et al. [8] compared the efficiency of flexible ureteroscopy and PCNL for treating kidney stones between 2 and 3 cm in a prospective matched study and demonstrated that the SFR was 76% and 87%, respectively, without a significant difference (*p* = 0.1). The complications were 27% and 29%, respectively (*p* = 0.4). However, the ureteroscopy group had shorter surgical times, shorter hospital stays and shorter convalescence but higher readmission rates than the nephrolithotomy group. Akman et al. [9] reported that the SFR after one session was 73.5% and 91.2% for RIRS and PCNL, respectively (*p* = 0.05), while the SFR in the RIRS group improved to 88.2% after a secondary procedure, which was not significantly different from PCNL.

A meta-analysis reviewed the literature for renal stones > 2 cm managed by ureteroscopy and holmium lasertripsy from 1990 to 2011 and showed that FURSL has a 95.7% stone-free rate with stones 2–3 cm and 84.6% in those > 3 cm, drawing the conclusion that FURSL can successfully treat patients with stones > 2 cm with a high stone-free rate and a low complication rate [10]. In the present study, FURSL was used to deal with upper pole calyceal calculi larger than 2 cm, and the initial SFR was 91.9%, which was in accordance with previous studies [1, 10]. The distinction in SFR may be explicable by the characteristics of the two procedures. The higher laser power and quick clearance across the percutaneous tract allow PNL to achieve stronger lithotripsy effectiveness and higher stone removal rates than FURSL. However, the time required for position change and placement of the ureter catheter accounted for a significant portion of PCNL's duration, making it longer than FURSL in our study.

It was reported that the overall incidence of complications of FURSL was 10.1%, while the incidence of severe complications was 0% ~ 5.3% [10]. Baş et al. [1] analysed 1359 cases of upper ureteral calculi and renal calculi treated with FURSL and found that the overall incidence of complications during operation was 5.9%, and the overall incidence of complications after operation was 7.3%. There were no severe complications in our present study, and the incidence of postoperative complications was 13.5%, which was much lower than that of PCNL. The RIRS group also had lower overall complication rates and shorter hospitalization times than the PCNL group. The risk of PCNL could not be disregarded while its character of high SFR. The surgery was associated with a higher incidence of complications, including bleeding, severe infection, and renal parenchymal damage. In the current study, intraoperative and postoperative complication rates were greater in the PNL group, particularly hemorrhage. This is also reflected in the drop levels of hemoglobin.

Despite its inferior SFR, FURSL has become a viable option for the treatment of the majority of kidney stones and its complication rates remain low. Nevertheless, rare fatal events, such as septic shock, may occur, especially in complex cases with a history of urinary tract infections [11]. Therefore, there are some tips to FURSL to improve the SFR of the procedure for upper pole calyceal calculi and maintain the safety of the operation. First, we recommend the Trendelenburg position for upper calyceal calculi to keep the stones as well as stone fragments thereafter in the upper calyx to avoid stone shifting. A UAS is also recommended for all FURSL procedures. It may ensure the circulation of flushing water, allow for optimal visualization, minimize resistance to the rotation of the ureteroscope and facilitate extraction of stone fragments. As an additional benefit, the sheath protects the ureter from repeated insertion and removal of the flexible ureteroscope [12]. However, it is difficult to place UAS in approximately 10% of patients due to ureteral stenosis [13]. For those patients, F6/7.5 ureteroscopic examination is recommended to check and dilate the ureter. If the stone burden is large or the patient cannot endure a long operation time, FURSL can be treated by several stages or combined with PCNL.

Second, in order to protect the ureteroscope, a high-power laser was not recommended, and a laser fibre too close to the lens was also not allowed. The flexible ureteroscope should be pulled back into the UAS when the holmium laser is being placed, adjusting the curvature of the ureteroscope and the angle of the optical fibre to bring the fibre close to the stones. During the operation, we used a low-frequency and high-energy strategy to break stones into fragments and then use a low-energy and high-frequency strategy for stone pulverization. Thus, during this series, we used a 365-μm holmium laser to break stones, and after extracting the larger fragments, the 200-μm holmium laser was then used for stone pulverization. Third, if the calculi are difficult to pulverize, a stone basket can be used to remove calculi over 2 mm to prevent steinstrasse and shorten the time of extraction. Finally, for patients with preoperative urinary infection, prophylactic antibiotics should be used, and percutaneous nephrostomy should be performed when necessary, which could be helpful to reduce intrarenal pressure and control the infection. Sustained low intra-pelvic pressure and application of UAS to shorten the operation time were conducive to reducing the incidence of postoperative infection [11].

FURSL offers several potential advantages in the management of large upper calyceal calculi. Using the natural route to the kidney reduces the degree of trauma and the risk of severe blood loss and possible irreversible loss of renal parenchyma; hence, it has low rates of severe haemorrhage, pleural injury and other complications. Furthermore, FURSL can save time and resources by avoiding the repositioning of the patient and the need to establish percutaneous access for prone PCNL [14, 15].

This research has certain drawbacks. This is a retrospective analysis conducted by a single institution. Our findings are based on a relatively limited number of samples. This strategy will also need to be examined with greater patient populations across multiple institutions.

## Conclusion

Both FURSL and mini-PNL are effective and safe for the management of upper calyceal calculi larger than 2 cm. Of these two procedures, FURSL is less time consuming, and FURSL is associated with faster recovery. FURSL can be considered a good alternative treatment in selected patients.

## Data Availability

The datasets generated and/or analysed during the current study are not publicly available due ethical restrctions but are available from the corresponding author on reasonable request.
